# *N*-(Fluoren-9-ylmethoxy­carbon­yl)-l-aspartic acid 4-*tert*-butyl ester

**DOI:** 10.1107/S1600536809037611

**Published:** 2009-10-03

**Authors:** Kazuhiko Yamada, Daisuke Hashizume, Tadashi Shimizu

**Affiliations:** a National Institute for Materials Science, 3-13 Sakura, Tsukuba 305-0003, Japan; b Advanced Technology Support Division, RIKEN, 2-1 Hirosawa, Wako, Saitama 351-0198, Japan

## Abstract

The bond distances and bond angles of the title compound, C_23_H_25_NO_6_, are consistent with values typically found for fluoren-9-ylmethoxy­carbonyl-protected amino acids. The conformations of the backbone and the side chain are slightly different from those of l-aspartic acid. The crystal structure exhibits two inter­molecular hydrogen bonds, forming a two-dimensional sheet structure parallel to the *ab* plane.

## Related literature

For the crystal structures of aspartic acids, see: Dawson (1977 ▶); Sequeira *et al.* (1989 ▶); Flaig *et al.* (1998 ▶); Rao (1973 ▶); Wang *et al.* (2007 ▶); Umadevi *et al.* (2003 ▶); Derissen *et al*. (1968 ▶); Bendeif & Jelsch (2007 ▶). For the crystal structures of *N*-α-fluoren-9-ylmethoxy­carbonyl-protected amino acids, see: Valle *et al.* (1984 ▶); Yamada, Hashizume & Shimizu (2008 ▶); Yamada, Hashizume, Shimizu & Deguchi (2008 ▶); Yamada, Hashizume, Shimizu, Ohiki & Yokoyama (2008 ▶).
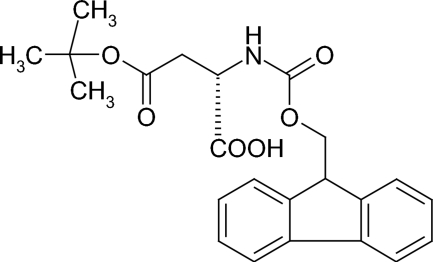


## Experimental

### 

#### Crystal data

C_23_H_25_NO_6_
*M* *_r_* = 411.44
Orthorhombic, 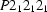

*a* = 5.7166 (4) Å
*b* = 11.1175 (10) Å
*c* = 32.083 (3) Å
*V* = 2039.0 (3) Å^3^
*Z* = 4
Mo *K*α radiation
μ = 0.10 mm^−1^
*T* = 90 K
0.11 × 0.05 × 0.04 mm

#### Data collection

Rigaku AFC-8 diffractometer with Saturn70 CCD detector
Absorption correction: none
15110 measured reflections
2722 independent reflections
2167 reflections with *I* > 2σ(*I*)
*R*_int_ = 0.077

#### Refinement

*R*[*F* ^2^ > 2σ(*F* ^2^)] = 0.059
*wR*(*F* ^2^) = 0.148
*S* = 1.13
2722 reflections
286 parameters
H-atom parameters constrained
Δρ_max_ = 0.29 e Å^−3^
Δρ_min_ = −0.27 e Å^−3^

### 

Data collection: *CrystalClear* (Rigaku/MSC, 2005 ▶); cell refinement: *HKL-2000* (Otwinowski & Minor, 1997 ▶); data reduction: *HKL-2000*; program(s) used to solve structure: *SIR2004* (Burla *et al.*, 2005 ▶); program(s) used to refine structure: *SHELXL97* (Sheldrick, 2008 ▶); molecular graphics: *ORTEP-3 for Windows* (Farrugia, 1997 ▶); software used to prepare material for publication: *SHELXL97*.

## Supplementary Material

Crystal structure: contains datablocks I, global. DOI: 10.1107/S1600536809037611/fj2243sup1.cif

Structure factors: contains datablocks I. DOI: 10.1107/S1600536809037611/fj2243Isup2.hkl

Additional supplementary materials:  crystallographic information; 3D view; checkCIF report

## Figures and Tables

**Table 1:** Hydrogen-bond geometry (Å, °)

*D*—H⋯*A*	*D*—H	H⋯*A*	*D*⋯*A*	*D*—H⋯*A*
O3—H3⋯O5^i^	0.84	1.91	2.744 (3)	172
N1—H1⋯O3^ii^	0.88	2.39	3.213 (4)	156

Symmetry codes: (i) 
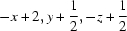
; (ii) 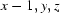.

**Fig. 1.**
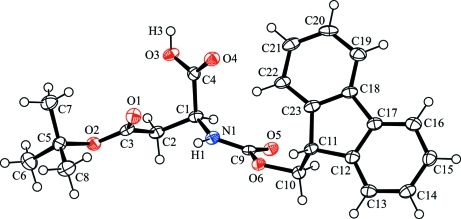
A view of the molecular structure of (I), showing the atom labeling scheme. Displacement ellipsoids are drawn at the 50% probability level.

**Fig. 2.**
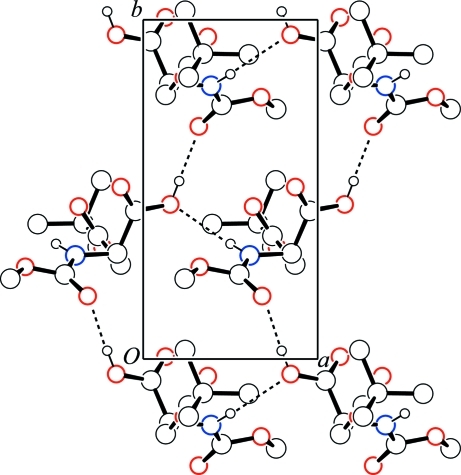
A packing diagram of (I) viewed from the c axis. Broken lines indicate the hydrogen bonds. The molecules in the region of 0 < z < 1/2 were plotted. The atoms of the fluoren-9-yl moiety and H atoms, except for H1 and H3, were omitted for clarity.

## supplementary crystallographic information

### Comment

L-Aspartic acid is one of the 20 building blocks of proteins, and, in mammals, can be produced from oxaloacetate by transamination. As for the related compounds of an aspartic acid, the crystal structures of L-aspartic acid (Derissen *et al.*,1968; Bendeif & Jelsch, 2007), L-aspartic acid monohydrate (Umadevi *et al.*, 2003), DL-aspartic acid (Sequeira *et al.*, 1989; Flaig *et al.*, 1998; Rao, 1973; Wang *et al.*, 2007), and DL-aspartic acid hydrochloride (Dawson, 1977) have been reported so far.

Fluoren-9-ylmethoxycarbonyl (Fmoc) group is widely used for solid-phase peptide synthesis protocols as an *N*-α-protecting group. To our best knowledge, however,there have been only four literatures reporting crystal structures of Fmoc-protected amino acids (Valle *et al.*, 1984; Yamada, Hashizume & Shimizu, 2008; Yamada, Hashizume, Shimizu & Deguchi, 2008; Yamada, Hashizume, Shimizu, Ohiki & Yokoyama, 2008). In this communication, the crystal structure of *N*-Fmoc-protected aspartic acid 4-*tert*-butyl ester (I) is reported.

The molecular structure of (I) is shown in Fig. 1 together with the atom labeling. The bond lengths and angles of the present molecule are in reasonable agreement with typical values found in L-aspartic acids and the related compounds. The conformations of the backbone and the side-chain, however, are slightly different from those of L-aspartic acid. The torsion angles, N1–C1–C2–C3 and N1–C1–C4–O4, are found to be 62.5 (4) and 17.0 (5)°, respectively. For L-aspartic acid, the corresponding angles are -60.3 and -39.2°, respectively. In the Fmoc-protected amino acids, the fluoren moiety takes various conformations as shown in the available literatures. In this case, the conformation of the Fmoc moiety is similar to those of Fmoc-protected isoleucine and serine.

Fig. 2 shows the crystal structure of (I). The molecules are linked *via* intermolecular hydrogen bonds between carboxyl and Fmoc moieties, O3–H3···O5 to form the column around the 2_1_ screw axis parallel to the *b* axis. The columns, related by translation symmeties along the *a* axis each other, are joined together through weak hydrogen bonds between the amino and carboxyl groups, N1—H1···O3, two-dimensional sheet structures are formed paralell to the *ab* plane consequentry. The geometries of the hydrogen bonds are listed in Table 2.

### Experimental

A powdered sample of the title compound was purchased from Wako Pure Chemical Industries, Ltd. (Osaka, Japan). Single crystals suitable for X-ray structure analysis could be obtained by recrystallization from ethyl acetate-dichloromethane (80:20) solution, which afforded white needle-like crystals.

### Refinement

All H atoms were located on the difference maps, and were treated as riding atoms with C/N/O–H distances of 1.00, 0.99, 0.98, 0.95, 0.88 and 0.84 Å, for methyne, methylene, methyl, phenyl, amino and hydroxyl groups, respectively, on the refinements. The *U*_iso_'s of the H atoms were fixed to be 1.2*U*_eq_(C/N) for methyne, methylene, phenyl and amino, or 1.5*U*_eq_(C/O) for methyl and hydroxyl of the parent atoms.

All Friedel pairs were merged, and all f"s of containing atoms were set to zero.

### Crystal data

**Table:**

C_23_H_25_NO_6_	*F*(000) = 872
*M**_r_* = 411.44	*D*_x_ = 1.340 Mg m^−^^3^
Orthorhombic, *P*2_1_2_1_2_1_	Mo *K*α radiation, λ = 0.71073 Å
Hall symbol: P 2ac 2ab	Cell parameters from 15181 reflections
*a* = 5.7166 (4) Å	θ = 1.8–27.6°
*b* = 11.1175 (10) Å	µ = 0.10 mm^−^^1^
*c* = 32.083 (3) Å	*T* = 90 K
*V* = 2039.0 (3) Å^3^	Needle, colourless
*Z* = 4	0.11 × 0.05 × 0.04 mm

### Data collection

**Table:**

Rigaku AFC-8 diffractometer with Saturn70 CCD detector	2167 reflections with *I* > 2σ(*I*)
Radiation source: fine-focus rotating anode	*R*_int_ = 0.077
confocal	θ_max_ = 27.6°, θ_min_ = 1.9°
Detector resolution: 28.5714 pixels mm^-1^	*h* = −7→6
ω scans	*k* = −14→10
15110 measured reflections	*l* = −41→41
2722 independent reflections	

### Refinement

**Table:**

Refinement on *F*^2^	Primary atom site location: structure-invariant direct methods
Least-squares matrix: full	Secondary atom site location: difference Fourier map
*R*[*F*^2^ > 2σ(*F*^2^)] = 0.059	Hydrogen site location: inferred from neighbouring sites
*wR*(*F*^2^) = 0.148	H-atom parameters constrained
*S* = 1.13	*w* = 1/[σ^2^(*F*_o_^2^) + (0.0694*P*)^2^ + 0.981*P*] where *P* = (*F*_o_^2^ + 2*F*_c_^2^)/3
2722 reflections	(Δ/σ)_max_ < 0.001
286 parameters	Δρ_max_ = 0.29 e Å^−^^3^
0 restraints	Δρ_min_ = −0.27 e Å^−^^3^

### Special details

**Table:**

Experimental. All Friedel pairs were merged, and all f"s of containing atoms were set to zero.
Geometry. All e.s.d.'s (except the e.s.d. in the dihedral angle between two l.s. planes) are estimated using the full covariance matrix. The cell e.s.d.'s are taken into account individually in the estimation of e.s.d.'s in distances, angles and torsion angles; correlations between e.s.d.'s in cell parameters are only used when they are defined by crystal symmetry. An approximate (isotropic) treatment of cell e.s.d.'s is used for estimating e.s.d.'s involving l.s. planes.
Refinement. Refinement of *F*^2^ against al reflections. The weighted *R*-factor *wR* and goodness of fit *S* are based on *F*^2^, conventional *R*-factors *R* are based on *F*, with *F* set to zero for negative *F*^2^. The threshold expression of *F*^2^ > 2σ(*F*^2^) is used only for calculating *R*-factors(gt) *etc*. and is not relevant to the choice of reflections for refinement. *R*-factors based on *F*^2^ are statistically about twice as large as those based on *F*, and *R*- factors based on all data will be even larger.

### Fractional atomic coordinates and isotropic or equivalent isotropic displacement parameters (Å^2^)

**Table:**

	*x*	*y*	*z*	*U*_iso_*/*U*_eq_	
O1	0.6301 (5)	0.4494 (2)	0.15286 (8)	0.0271 (6)	
O2	0.7666 (5)	0.3298 (2)	0.10086 (7)	0.0237 (6)	
O3	1.1545 (5)	0.4557 (2)	0.20322 (8)	0.0254 (6)	
H3	1.2064	0.5245	0.2089	0.038*	
O4	0.8720 (5)	0.5069 (2)	0.24884 (8)	0.0267 (6)	
O5	0.6813 (5)	0.1857 (2)	0.28643 (7)	0.0230 (6)	
O6	0.3240 (5)	0.2717 (2)	0.27449 (7)	0.0207 (6)	
N1	0.6108 (6)	0.3053 (3)	0.22971 (9)	0.0221 (7)	
H1	0.5004	0.3395	0.2147	0.027*	
C1	0.8504 (7)	0.3115 (3)	0.21511 (11)	0.0203 (8)	
H1A	0.9431	0.2511	0.2313	0.024*	
C2	0.8711 (7)	0.2786 (3)	0.16889 (11)	0.0221 (8)	
H2A	1.0385	0.2787	0.1610	0.027*	
H2B	0.8106	0.1961	0.1648	0.027*	
C3	0.7406 (7)	0.3629 (3)	0.14049 (11)	0.0205 (8)	
C4	0.9549 (7)	0.4366 (3)	0.22432 (11)	0.0215 (8)	
C5	0.6574 (8)	0.4001 (3)	0.06656 (11)	0.0254 (9)	
C6	0.7397 (9)	0.3306 (4)	0.02814 (12)	0.0363 (11)	
H6A	0.6839	0.2474	0.0298	0.054*	
H6B	0.9110	0.3312	0.0270	0.054*	
H6C	0.6768	0.3687	0.0030	0.054*	
C7	0.7598 (8)	0.5265 (3)	0.06653 (13)	0.0309 (9)	
H7A	0.9308	0.5216	0.0676	0.046*	
H7B	0.7024	0.5706	0.0909	0.046*	
H7C	0.7120	0.5685	0.0411	0.046*	
C8	0.3951 (8)	0.3978 (4)	0.07039 (14)	0.0333 (10)	
H8A	0.3481	0.4396	0.0959	0.050*	
H8B	0.3410	0.3142	0.0715	0.050*	
H8C	0.3253	0.4381	0.0462	0.050*	
C9	0.5502 (7)	0.2491 (3)	0.26529 (11)	0.0180 (7)	
C10	0.2379 (7)	0.2366 (3)	0.31542 (10)	0.0206 (8)	
H10A	0.3663	0.2015	0.3322	0.025*	
H10B	0.1125	0.1758	0.3126	0.025*	
C11	0.1424 (7)	0.3503 (3)	0.33673 (11)	0.0199 (8)	
H11	0.0232	0.3901	0.3185	0.024*	
C12	0.0379 (7)	0.3219 (3)	0.37912 (11)	0.0231 (8)	
C13	−0.1547 (8)	0.2505 (3)	0.38905 (11)	0.0243 (8)	
H13	−0.2397	0.2099	0.3679	0.029*	
C14	−0.2195 (7)	0.2401 (3)	0.43060 (12)	0.0263 (9)	
H14	−0.3507	0.1918	0.4378	0.032*	
C15	−0.0960 (8)	0.2990 (3)	0.46202 (12)	0.0284 (9)	
H15	−0.1433	0.2903	0.4902	0.034*	
C16	0.0969 (7)	0.3707 (3)	0.45224 (12)	0.0261 (9)	
H16	0.1830	0.4103	0.4735	0.031*	
C17	0.1598 (8)	0.3825 (3)	0.41070 (11)	0.0227 (8)	
C18	0.3500 (7)	0.4533 (3)	0.39111 (11)	0.0220 (8)	
C19	0.5228 (8)	0.5253 (3)	0.40880 (12)	0.0286 (9)	
H19	0.5306	0.5365	0.4381	0.034*	
C20	0.6843 (8)	0.5807 (3)	0.38242 (13)	0.0302 (10)	
H20	0.8067	0.6279	0.3940	0.036*	
C21	0.6685 (8)	0.5678 (3)	0.33946 (13)	0.0289 (9)	
H21	0.7788	0.6072	0.3220	0.035*	
C22	0.4919 (8)	0.4973 (3)	0.32146 (12)	0.0250 (8)	
H22	0.4792	0.4901	0.2920	0.030*	
C23	0.3357 (7)	0.4382 (3)	0.34766 (11)	0.0230 (8)	

### Atomic displacement parameters (Å^2^)

**Table:**

	*U*^11^	*U*^22^	*U*^33^	*U*^12^	*U*^13^	*U*^23^
O1	0.0316 (17)	0.0205 (13)	0.0293 (14)	0.0069 (13)	0.0030 (13)	0.0004 (10)
O2	0.0291 (16)	0.0162 (12)	0.0258 (13)	0.0015 (12)	−0.0022 (12)	0.0003 (10)
O3	0.0280 (16)	0.0139 (12)	0.0342 (14)	−0.0014 (12)	0.0038 (13)	0.0000 (10)
O4	0.0299 (17)	0.0195 (12)	0.0306 (13)	0.0004 (13)	0.0039 (13)	−0.0027 (11)
O5	0.0225 (14)	0.0157 (12)	0.0307 (13)	0.0032 (11)	0.0002 (12)	0.0030 (10)
O6	0.0205 (14)	0.0179 (12)	0.0237 (12)	−0.0001 (11)	−0.0007 (11)	0.0004 (9)
N1	0.0208 (18)	0.0186 (15)	0.0269 (15)	0.0018 (14)	−0.0007 (13)	0.0030 (12)
C1	0.0191 (19)	0.0139 (16)	0.0279 (18)	0.0013 (16)	−0.0016 (16)	0.0020 (13)
C2	0.024 (2)	0.0115 (16)	0.0305 (18)	0.0027 (15)	0.0019 (17)	0.0004 (13)
C3	0.0168 (19)	0.0160 (17)	0.0286 (19)	−0.0019 (15)	0.0021 (16)	0.0032 (14)
C4	0.023 (2)	0.0199 (17)	0.0212 (17)	0.0022 (16)	−0.0020 (16)	0.0023 (14)
C5	0.029 (2)	0.0225 (18)	0.0248 (18)	−0.0006 (17)	−0.0020 (19)	0.0034 (14)
C6	0.041 (3)	0.037 (2)	0.031 (2)	0.003 (2)	−0.001 (2)	−0.0055 (18)
C7	0.031 (2)	0.0233 (19)	0.039 (2)	0.0000 (18)	−0.003 (2)	0.0060 (16)
C8	0.024 (2)	0.031 (2)	0.045 (2)	−0.0005 (18)	−0.007 (2)	0.0090 (19)
C9	0.0171 (18)	0.0119 (16)	0.0249 (17)	−0.0016 (14)	−0.0015 (15)	−0.0022 (13)
C10	0.0212 (19)	0.0169 (16)	0.0236 (17)	0.0028 (15)	0.0000 (16)	0.0004 (13)
C11	0.021 (2)	0.0133 (16)	0.0260 (17)	0.0008 (16)	0.0001 (17)	0.0016 (13)
C12	0.028 (2)	0.0135 (16)	0.0278 (18)	0.0033 (16)	0.0004 (17)	0.0006 (14)
C13	0.025 (2)	0.0162 (17)	0.0316 (19)	0.0022 (17)	−0.0024 (18)	−0.0024 (14)
C14	0.024 (2)	0.0176 (17)	0.037 (2)	0.0013 (16)	0.0061 (18)	−0.0002 (15)
C15	0.038 (3)	0.0202 (19)	0.0270 (19)	0.0022 (18)	0.0056 (18)	0.0016 (14)
C16	0.030 (2)	0.0175 (18)	0.031 (2)	0.0028 (17)	−0.0008 (18)	−0.0040 (14)
C17	0.024 (2)	0.0139 (16)	0.0305 (19)	0.0019 (16)	−0.0001 (17)	−0.0002 (13)
C18	0.020 (2)	0.0140 (16)	0.0317 (19)	0.0006 (16)	0.0002 (17)	−0.0017 (13)
C19	0.034 (3)	0.0188 (18)	0.033 (2)	−0.0009 (17)	−0.0024 (19)	−0.0046 (15)
C20	0.030 (2)	0.0152 (18)	0.045 (2)	−0.0057 (17)	−0.001 (2)	−0.0088 (15)
C21	0.030 (2)	0.0138 (17)	0.043 (2)	−0.0010 (17)	0.008 (2)	−0.0010 (15)
C22	0.029 (2)	0.0131 (16)	0.0326 (19)	−0.0014 (17)	0.0021 (18)	−0.0015 (14)
C23	0.022 (2)	0.0122 (16)	0.035 (2)	0.0020 (16)	0.0013 (18)	0.0005 (13)

### Geometric parameters (Å, °)

**Table:**

O1—C3	1.218 (4)	C8—H8C	0.9800
O2—C3	1.332 (4)	C10—C11	1.537 (5)
O2—C5	1.487 (4)	C10—H10A	0.9900
O3—C4	1.344 (5)	C10—H10B	0.9900
O3—H3	0.8400	C11—C23	1.516 (5)
O4—C4	1.206 (4)	C11—C12	1.518 (5)
O5—C9	1.232 (4)	C11—H11	1.0000
O6—C9	1.350 (5)	C12—C13	1.395 (6)
O6—C10	1.456 (4)	C12—C17	1.402 (5)
N1—C9	1.346 (4)	C13—C14	1.388 (5)
N1—C1	1.450 (5)	C13—H13	0.9500
N1—H1	0.8800	C14—C15	1.394 (6)
C1—C2	1.532 (5)	C14—H14	0.9500
C1—C4	1.542 (5)	C15—C16	1.397 (6)
C1—H1A	1.0000	C15—H15	0.9500
C2—C3	1.504 (5)	C16—C17	1.387 (5)
C2—H2A	0.9900	C16—H16	0.9500
C2—H2B	0.9900	C17—C18	1.482 (5)
C5—C8	1.504 (6)	C18—C19	1.392 (5)
C5—C7	1.522 (5)	C18—C23	1.407 (5)
C5—C6	1.529 (5)	C19—C20	1.395 (6)
C6—H6A	0.9800	C19—H19	0.9500
C6—H6B	0.9800	C20—C21	1.389 (6)
C6—H6C	0.9800	C20—H20	0.9500
C7—H7A	0.9800	C21—C22	1.402 (6)
C7—H7B	0.9800	C21—H21	0.9500
C7—H7C	0.9800	C22—C23	1.392 (5)
C8—H8A	0.9800	C22—H22	0.9500
C8—H8B	0.9800		
			
C3—O2—C5	120.9 (3)	N1—C9—O6	110.2 (3)
C4—O3—H3	109.5	O6—C10—C11	107.5 (3)
C9—O6—C10	118.1 (3)	O6—C10—H10A	110.2
C9—N1—C1	122.6 (3)	C11—C10—H10A	110.2
C9—N1—H1	118.7	O6—C10—H10B	110.2
C1—N1—H1	118.7	C11—C10—H10B	110.2
N1—C1—C2	112.0 (3)	H10A—C10—H10B	108.5
N1—C1—C4	110.3 (3)	C23—C11—C12	102.3 (3)
C2—C1—C4	111.7 (3)	C23—C11—C10	112.0 (3)
N1—C1—H1A	107.5	C12—C11—C10	111.5 (3)
C2—C1—H1A	107.5	C23—C11—H11	110.3
C4—C1—H1A	107.5	C12—C11—H11	110.3
C3—C2—C1	113.6 (3)	C10—C11—H11	110.3
C3—C2—H2A	108.9	C13—C12—C17	120.1 (3)
C1—C2—H2A	108.9	C13—C12—C11	129.3 (3)
C3—C2—H2B	108.9	C17—C12—C11	110.6 (3)
C1—C2—H2B	108.9	C14—C13—C12	118.5 (4)
H2A—C2—H2B	107.7	C14—C13—H13	120.7
O1—C3—O2	126.0 (3)	C12—C13—H13	120.7
O1—C3—C2	123.5 (3)	C13—C14—C15	121.3 (4)
O2—C3—C2	110.5 (3)	C13—C14—H14	119.3
O4—C4—O3	124.1 (3)	C15—C14—H14	119.3
O4—C4—C1	123.8 (4)	C14—C15—C16	120.4 (4)
O3—C4—C1	112.0 (3)	C14—C15—H15	119.8
O2—C5—C8	110.4 (3)	C16—C15—H15	119.8
O2—C5—C7	108.9 (3)	C17—C16—C15	118.4 (4)
C8—C5—C7	113.5 (4)	C17—C16—H16	120.8
O2—C5—C6	101.6 (3)	C15—C16—H16	120.8
C8—C5—C6	111.3 (4)	C16—C17—C12	121.3 (4)
C7—C5—C6	110.3 (3)	C16—C17—C18	130.4 (4)
C5—C6—H6A	109.5	C12—C17—C18	108.3 (3)
C5—C6—H6B	109.5	C19—C18—C23	120.9 (4)
H6A—C6—H6B	109.5	C19—C18—C17	130.8 (3)
C5—C6—H6C	109.5	C23—C18—C17	108.3 (3)
H6A—C6—H6C	109.5	C18—C19—C20	118.4 (4)
H6B—C6—H6C	109.5	C18—C19—H19	120.8
C5—C7—H7A	109.5	C20—C19—H19	120.8
C5—C7—H7B	109.5	C21—C20—C19	120.9 (4)
H7A—C7—H7B	109.5	C21—C20—H20	119.6
C5—C7—H7C	109.5	C19—C20—H20	119.6
H7A—C7—H7C	109.5	C20—C21—C22	120.9 (4)
H7B—C7—H7C	109.5	C20—C21—H21	119.6
C5—C8—H8A	109.5	C22—C21—H21	119.6
C5—C8—H8B	109.5	C23—C22—C21	118.5 (4)
H8A—C8—H8B	109.5	C23—C22—H22	120.7
C5—C8—H8C	109.5	C21—C22—H22	120.7
H8A—C8—H8C	109.5	C22—C23—C18	120.3 (4)
H8B—C8—H8C	109.5	C22—C23—C11	129.2 (3)
O5—C9—N1	125.1 (4)	C18—C23—C11	110.4 (3)
O5—C9—O6	124.7 (3)		
			
C9—N1—C1—C2	132.6 (3)	C12—C13—C14—C15	−0.2 (6)
C9—N1—C1—C4	−102.3 (4)	C13—C14—C15—C16	0.2 (6)
N1—C1—C2—C3	62.5 (4)	C14—C15—C16—C17	0.7 (6)
C4—C1—C2—C3	−61.9 (4)	C15—C16—C17—C12	−1.6 (6)
C5—O2—C3—O1	0.3 (6)	C15—C16—C17—C18	178.6 (4)
C5—O2—C3—C2	−179.0 (3)	C13—C12—C17—C16	1.7 (6)
C1—C2—C3—O1	0.5 (5)	C11—C12—C17—C16	−179.9 (4)
C1—C2—C3—O2	179.7 (3)	C13—C12—C17—C18	−178.5 (3)
N1—C1—C4—O4	17.0 (5)	C11—C12—C17—C18	−0.1 (4)
C2—C1—C4—O4	142.3 (4)	C16—C17—C18—C19	1.9 (7)
N1—C1—C4—O3	−165.8 (3)	C12—C17—C18—C19	−177.9 (4)
C2—C1—C4—O3	−40.5 (4)	C16—C17—C18—C23	−178.1 (4)
C3—O2—C5—C8	−63.1 (4)	C12—C17—C18—C23	2.1 (4)
C3—O2—C5—C7	62.2 (5)	C23—C18—C19—C20	−1.0 (6)
C3—O2—C5—C6	178.7 (3)	C17—C18—C19—C20	178.9 (4)
C1—N1—C9—O5	−9.2 (5)	C18—C19—C20—C21	2.2 (6)
C1—N1—C9—O6	171.1 (3)	C19—C20—C21—C22	−1.0 (6)
C10—O6—C9—O5	10.9 (5)	C20—C21—C22—C23	−1.5 (6)
C10—O6—C9—N1	−169.3 (3)	C21—C22—C23—C18	2.6 (6)
C9—O6—C10—C11	122.3 (3)	C21—C22—C23—C11	−175.1 (4)
O6—C10—C11—C23	−68.6 (4)	C19—C18—C23—C22	−1.4 (6)
O6—C10—C11—C12	177.4 (3)	C17—C18—C23—C22	178.7 (3)
C23—C11—C12—C13	176.5 (4)	C19—C18—C23—C11	176.7 (3)
C10—C11—C12—C13	−63.7 (5)	C17—C18—C23—C11	−3.2 (4)
C23—C11—C12—C17	−1.7 (4)	C12—C11—C23—C22	−179.1 (4)
C10—C11—C12—C17	118.1 (4)	C10—C11—C23—C22	61.3 (5)
C17—C12—C13—C14	−0.8 (5)	C12—C11—C23—C18	3.0 (4)
C11—C12—C13—C14	−178.8 (4)	C10—C11—C23—C18	−116.5 (3)

### Hydrogen-bond geometry (Å, °)

**Table:**

*D*—H···*A*	*D*—H	H···*A*	*D*···*A*	*D*—H···*A*
O3—H3···O5^i^	0.84	1.91	2.744 (3)	172
N1—H1···O3^ii^	0.88	2.39	3.213 (4)	156

Symmetry codes: (i) −*x*+2, *y*+1/2, −*z*+1/2; (ii) *x*−1, *y*, *z*.
